# Podoplanin-positive dilated lymphatic vessels in duodenum associates with three-month mortality in patients with cirrhosis

**DOI:** 10.3389/fphys.2023.1045983

**Published:** 2023-05-25

**Authors:** Pinky Juneja, Aarti Sharma, S. M. Shasthry, Guresh Kumar, Dinesh M. Tripathi, V. Rajan, Archana Rastogi, Shiv K. Sarin, Savneet Kaur

**Affiliations:** ^1^ Department of Molecular and Cellular Medicine, Institute of Liver and Biliary Sciences, New Delhi, India; ^2^ Department of Hepatology, Institute of Liver and Biliary Sciences, New Delhi, India; ^3^ Department of Pathology, Institute of Liver and Biliary Sciences, New Delhi, India

**Keywords:** cirrhosis, gut lymphatic vessels, duodenal biopsy, podoplanin, inflammation

## Abstract

Dilated and dysfunctional gut lymphatic vessels (LVs) have been reported in experimental cirrhosis. Here, we studied LVs in duodenal (D2)-biopsies of liver cirrhosis patients and investigated the prognostic role of a LV marker, podoplanin (PDPN), in predicting the mortality of patients with cirrhosis. A prospective, single-center cohort study was performed in liver cirrhosis patients (*n* = 31) and matched healthy controls (*n* = 9). D2-biopsies were obtained during endoscopy procedure, immunostained with PDPN, and scored based on 1) intensity and 2) density of positively-stained LVs per high power field. Gut and systemic inflammation were estimated by quantifying duodenal CD3^+^ intraepithelial lymphocytes (IELs), CD68^+^ macrophages, and serum TNF-α and IL-6 levels, respectively. Gut permeability and inflammation as assessed by quantifying gene expression of *TJP1*, *OCLN, TNF-α,* and *IL-6* in D2-biopsies. Gene expression of LV markers, *PDPN* (8-fold), and *LYVE1* (3-fold) was enhanced in D2-biopsies of cirrhosis patients compared to control (*p* < 0.0001). The mean PDPN score in decompensated cirrhosis patients (6.91 ± 1.26, *p* < 0.0001) was significantly increased as compared to those with compensated (3.25 ± 1.60). PDPN score positively and significantly correlated with the number of IELs (r = 0.33), serum TNF-α (r = 0.35), and IL-6 (r = 0.48) levels, while inversely correlated with *TJP1* expression (r = -0.46, *p* < 0.05 each). In Cox regression, the PDPN score was a significant and independent 3-month-mortality predictor in patients (HR: 5.61; 1.08-29.109; *p* = 0.04). The area under the curve for the PDPN score was 84.2, and cutoff value for predicting mortality was ≥6.5 with 100% sensitivity and 75% specificity. Collectively, dilated LVs with high PDPN expression in D2-biopsies is a characteristic feature of patients with decompensated cirrhosis. PDPN score correlates with enhanced gut and systemic inflammation and also associates with 3-month mortality in cirrhosis.

## 1 Introduction

The intestinal lymphatic vasculature comprises the longest-studied lymphatic vessel (LVs) bed. It plays a major role in dietary fat uptake and transport, viscera fluid balance, and gut immunosurveillance (Bernier-Latmani and Petrova, 2017). The intestinal lymphatic capillaries (lacteals) and associated collecting vessels in the mesentery form the drainpipe that clears interstitial fluid, inflammatory cells and molecules, and microbial debris from the abdominal viscera. The intestinal lymphatic system has been associated with pathologies, including inflammatory bowel disease and obesity (D’alessio et al., 2014; Cao et al., 2021). Intestinal inflammation is correlated with extensive intestinal lymphangiogenesis, and specific loss of intestinal lymphatics results in severe gut inflammation, sepsis, and complete short-term lethality, highlighting the crucial role of intestinal lymphatics in inflammation (Jang et al., 2013).

In patients with cirrhosis and portal hypertension, studies have demonstrated a significant increase in abdominal lymph production and lymph flow in thoracic duct (Vollmar et al., 1997). Using rat model of CCl4-induced cirrhosis, Ribera et al. documented gut LVs remodeling and impaired drainage, characterized by a decline of smooth muscle cells and, hence, its contractile activity due to increased endothelial expression nitric oxide synthase in mesenteric vessels (Ribera et al., 2013). However, no studies have characterized gut LVs in clinical studies. An understanding of intestinal LVs in patients with cirrhosis might lead us to novel therapeutic strategies. To this end, we thoroughly investigated duodenal (D2) LVs in patients with liver cirrhosis and studied their association with disease severity, inflammatory markers, and survival.

## 2 Methods

### 2.1 Study design and patients

We performed a prospective, observational, single-center cohort study in about 6 months in patients with histologically confirmed cirrhosis (viral, alcoholic, or other aetiology) and clinical parameters from the Department of Hepatology of Institute of Liver and Biliary Sciences, listed in Table 1. We excluded patients with active bacterial infection at evaluation, hepatocellular carcinoma, active alcohol abuse, variceal bleed (4 weeks), recent previous transjugular intrahepatic portosystemic shunt insertion, occlusive portal vein thrombosis, liver transplantation (LT), chronic kidney disease, gastrointestinal mucosal diseases (e.g., celiac disease, inflammatory bowel disease) or intestinal surgery. Patients with cirrhosis were subsequently divided into two groups: decompensated (*n* = 19) or compensated (*n* = 12) cirrhosis based on the presence or history of at least one decompensating event, i.e., ascites, jaundice, variceal bleeding, and overt hepatic encephalopathy (HE). The control group (*n* = 9) comprised individuals without any liver disease matched for age and sex which underwent an upper gastrointestinal tract endoscopy for non-cirrhotic causes, e.g., functional dyspepsia. All subjects enrolled in study underwent an upper gastrointestinal tract endoscopy for clinical indications, during which biopsies were obtained from second portion of duodenum, distal to the ampulla of Vater, after their consent before the study. The study was conducted according to the principles of the Declaration of Helsinki and was approved by the institutional ethics committee (IEC/2019/68/NA05). All patients gave written informed consent for participation.

**TABLE 1:** Demographic and clinical characteristics of the patients (N = 40).

Parameters	Controls (N = 9)	Compensated (N = 12)	Decompensated (N = 19)	*p*-Value (all 3 groups)	*p*-Value
					Comp vs. decomp
Age (years)	48 ± 11.9	52 ± 10.5	52 ± 9.49	NS	NS
Male N (%)	6 (60)	8 (66.6)	17 (89.4)	NS	NS
Platelet count (x 10^9^/L)	254.1 ± 84.43	172.5 ± 57.11	117.4 ± 48.2	<0.0001	0.02
Bilirubin (mg/dl)	0.6 ± 0.27	1.4 ± 0.95	5.1 ± 6.71	0.03	0.07
Aspartate transaminases (AST*, U/L)	27.5 (20.3–44)	39 (28–72)	60 (24–221)	0.01	0.04
Alanine transaminase (ALT*, U/L)	28.85 (19–47)	30.50 (21–80)	33 (20–144)	NS	NS
Albumin (g/dl)	4.2 ± 0.64	3.7 ± 0.79	3.1 ± 0.56	0.0006	0.01
Serum Sodium (mEq/L)	138.2 ± 3.06	137.8 ± 3.72	131.7 ± 4.30	NS	NS
INR	1.1 ± 0.12	1.3 ± 0.22	1.6 ± 0.59	0.006	0.04
Creatinine (mg/dl)	0.9 ± 0.16	0.7 ± 0.19	1.3 ± 0.74	0.01	0.01
Etiology of liver diseases n (%)					
Alcohol N (%)		3 (25)	9 (47.3)		NS
HBV N (%)		1 (8.3)	2 (10.5)		NS
HCV N (%)		0	0		
NASH N (%)		7 (58.3)	7 (36.8)		NS
Others N (%)		1 (8.3)	1 (5.2)		NS
Ascites N (%)		0	19 (100)		-
Hepatic encephalopathy N (%)		0	9 (47.3)		-
Variceal Bleeding N (%)		0	8 (42.1)		-
Esophageal Varices N (%)		1 (8.3)	14 (73.6)		0.0004
Liver-Related 3-month mortality N (%)		0	7 (36.8)		-
CTP Score	-	5.67 ± 2.8	9.1 ± 2.06	<0.0001	<0.0001
MELD score	-	8.3 ± 5.4	18.6 ± 6.41	<0.0001	<0.0001

For continuous variables, values are given as mean (SD) or Median (min-max), and *p* values have been calculated by Student’s ‘t' test or Mann-Whitney U test. Categorical variables are given as N (%), and *p* values for categorical variables have been calculated using Fisher exact test. HBV: Hepatitis B Virus; HCV: Hepatitis C Virus; NASH: Non-alcoholic steatohepatitis; CTP: Child-Turcotte-Pugh; MELD: a model for end-stage liver disease.

In addition, routine laboratory tests for liver dysfunction severity were measured, e.g., Model for End-Stage Liver Disease (MELD) and Child-Pugh scores (CTP). Along with this, *TNF-α* and *IL-6* mRNA and their serum levels were assessed as biomarkers of gut and systemic inflammation by ELISA following the manufacturer’s instructions. Commercial ELISA kits were used to measure the biomarker levels (E-lab-sciences, Unites States). All the clinical and biochemical investigations were done at the time of biopsy collection. After collecting the D2-biopsy, patients were followed for 3 months to record mortality.

### 2.2 RNA isolation and RT-PCRs

D2-biopsies were snap-frozen in liquid nitrogen, and total RNA was isolated from the TRIzol reagent (Thermofisher, India). RNA was quantified with Thermo Scientific Nanodrop 2,000 Spectrophotometer. According to the manufacturer’s instructions, cDNA was synthesized using 1 μg of RNA with reverse transcriptase (Thermo Revert aid cDNA synthesis kit). qRT-PCR was performed with SYBR green PCR master mix (Fermentas Life Sciences) on the ViiA7 PCR system (Applied Biosystems, United States). The cycling parameters used were as follows: start at 95°C for 5 min, denaturing at 95°C for 30 s, annealing at 60°C for 30 s, elongation at 72°C for 30 s, and final 5 min extra extension at the end of the reaction and repeated for 40 amplification cycles. After normalization, relative quantification of the expression was done using the ΔΔCt method to the expression of the housekeeping gene, GAPDH. The genes and primer pairs are mentioned in Supplementary Table S1.

### 2.3 Immunohistochemistry

D2-biopsies were fixed in 10% buffered formalin and processed. Sections of 4-μm-thick paraffin-embedded tissues were heat-fixed, deparaffinized at 45 C, and rehydrated in descending ethanol series. Following antigen retrieval by heating for 8 min in a microwave with citrate buffer, pH 6, sections were incubated for 20 min with peroxidase-1 solution to quench endogenous peroxidase. Protein blocking was done with 3% BSA. Slides were then incubated overnight at 4 C in the humid chamber with primary antibodies (Supplementary Table S2). Staining was completed using the HRP-conjugated mouse/rat/human detection kit and DAB chromogen as a substrate, according to the manufacturer’s instructions. Lastly, sections were counterstained with hematoxylin. Slides were mounted with a coverslip using DPX (Dibutylphthalate Polystyrene Xylene). LVs were semi-quantitatively quantified based on the expression of podoplanin (PDPN), a surface marker present on lymphatic endothelium. The total PDPN score was the sum of 1) the intensity of stained area measured in percentage using ImageJ [0- none; 1 (1%–25%); 2 (25%–50%); 3 (50%–75%); 4 (75%–100%)] and 2) the percentage proportion of PDPN + stained area/field [0 (0%–5%); 1 (6%–25%); 2 (26%–50%), and 3 (51%–75%) and 4 (76%–100%)] (Klein et al., 2001) (Supplementary Figure S1, Supplementary Table S3). Scores between 0 and 4 were considered to indicate low PDPN expression, while scores of five to eight represented high expression. Pathologists, blinded to clinicopathological details of patients and identity of slides, conducted IHC analysis. PDPN scores in each sample were based on analysis of six randomly selected fields per slide. The diameter of LVs was quantified using ImageJ. Each image was calibrated as per the scale bar, and the diameter was measured in PDPN-stained LVs. Per slide, six random fields were selected for analysis. For scoring intraepithelial lymphocytes (IELs) and macrophages, D2-biopsy sections were stained with ready-to-use CD3 and CD68 anti-human antibodies (PathnSitu Biotechnologies), respectively. According to the manufacturer’s instructions, the staining was completed using the PathnSitu HRP-conjugated detection kit and DAB chromogen as a substrate and counterstained with hematoxylin. For the quantification of CD3^+^ and CD68^+^ cells, six fields per slide were selected. CD3^+^ IELs were counted per 100 epithelial cells, and less than 30 IELs were considered normal. CD68^+^ cells were quantified per field. For VEGF-C expression, slides were stained with VEGF-C (VEGF-C antibody, MA5-26494, Invitrogen, Unites States, 1:100 dilution). Six random fields per slide were selected. Mean staining density was determined using ImageJ (ImageJ, NIH, United States), and pathologists interpreted staining results.

### 2.5 Statistical analysis

Categorical variables were reported as absolute frequencies n) and relative frequencies (%); continuous variables as mean ± SD. Normal distribution was assessed by Kolmogorov-Smirnov tests. Categorical data were analyzed with the Chi-square or Fisher’s exact test wherever appropriate. Continuous variables were compared with an independent sample *t*-test or Mann-Whitney U test for two groups and by One-way ANOVA or Kruskal–Wallis’s test for more than two groups depending on the distribution (normal or skewed). For the assessment of prognostic factors in decompensated cirrhosis, clinical predictors were analyzed by binary logistic regression as the time of decompensation was not available. For analysis of mortality predictors, all clinical variables were entered into the Cox hazard regression model to assess the effects of factors on 3-month mortality from the date of biopsy collection. We used unadjusted linear regression models and multivariate models adjusted for clinical covariates. The receiver operating characteristic (ROC) curves were plotted to explore the area under the curve (AUC). Cutoff points to discriminate the survivors from the non-survivors were calculated by obtaining the best Youden index (sensitivity% + specificity% − 100). The cutoff values were taken where sensitivity and specificity were optimal.

AUCs were expressed with their 95% confidence interval (CI). The study participants were divided into two groups according to the optimal cutoff value of 3-month mortality/LT probability. Then the mortality rates of the groups were compared using Kaplan–Meier curve analysis. A log-rank test was conducted to compare the survival curves of the groups. Correlation analysis between the variables was performed by using Pearson’s Correlation analysis. All statistical analyses were performed using IBM SPSS Statistics (version 20.0, SPSS Inc., Chicago, IL, United States) and GraphPad Prism 8 (GraphPad Software, CA, United States). Statistical analysis was done using GraphPad Prism (version Prism 8.4.3; GraphPad Software, San Diego, CA, United States) and SPSS. The level of significance was set at a 2-sided *p*-value < 0.05.

## 3 Results

### 3.1 Demographic features of the study groups

Characteristics of the patient groups are summarized in Table 1. The mean age was comparable between the study groups, with 48 ± 11.9 years for controls, 52 ± 10.5, and 52 ± 9.49 for compensated and decompensated cirrhotic patients. The control group was significantly different from the cirrhotic patients with respect to the clinical variables. Decompensated cirrhotic had lower platelet and serum albumin levels and higher serum bilirubin, AST, INR, and creatinine compared to compensated cirrhosis (*p* < 0.05, Table 1). Non-alcoholic fatty liver disease (45.1%) and alcohol-related liver disease (38.7%) were the most common etiologies for liver cirrhosis in the study cohort. Among patients with decompensated cirrhosis, 19 (100%) had ascites, 8 (42.1%) had variceal bleeding, and 9 (47.3%) had hepatic encephalopathy. The CTP and MELD scores were significantly higher in decompensated *versus* compensated cirrhosis (*p* < 0.0001 for both, Table 1).

### 3.2 Enhanced PDPN+ dilated lymphatic vessels density in decompensated cirrhosis

**FIGURE 1:**
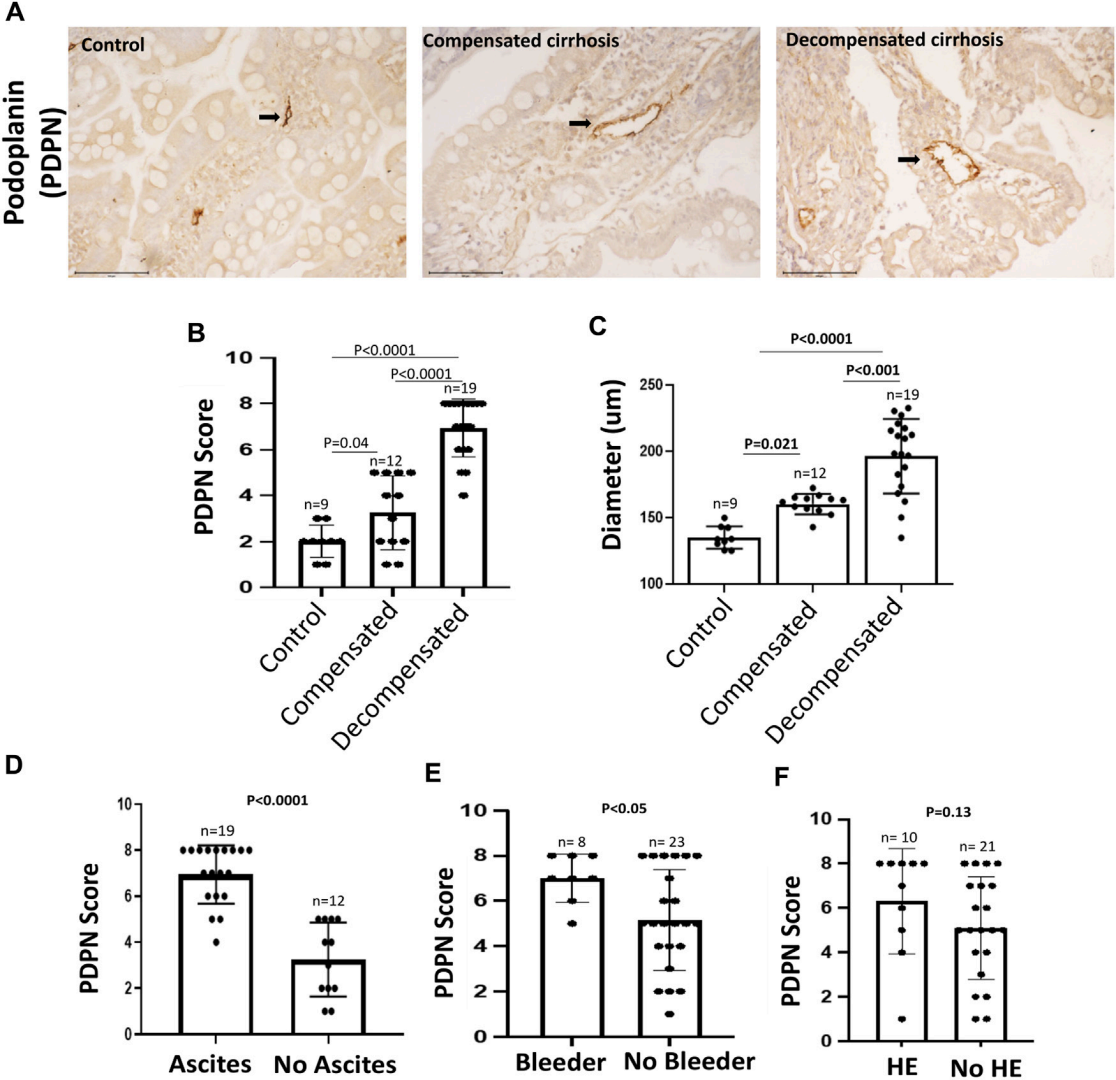
PDPN score in different stages of cirrhosis and associated complications **(A)** Representative PDPN immunostained sections of D2-biopsies in control, patient with compensated and decompensated cirrhosis. Scale bar: 100 µM. Bar graph showing **(B)** PDPN score and **(C)** Diameter of LVs in duodenum biopsy of control (*n* = 9), compensated (*n* = 12), and decompensated (*n* = 19) cirrhotic patients. Bar graph showing PDPN score in patients with compensated and decompensated cirrhosis **(D)** with (*n* = 19) and without (*n* = 12) ascites **(E)** with (*n* = 8) and without (*n* = 23) variceal bleeding, and **(F)** with (*n* = 10) and without (*n* = 21) hepatic encephalopathy (HE). Data represent mean ± standard deviation. Differences between groups were calculated by students’ unpaired ‘*t*-test. LVs: Lymphatic vessels; D2: Duodenal.

An impaired gut lymphatic system in terms of increased density, dilation, leakage, and reduced drainage has been reported previously in experimental liver cirrhosis with ascites (Ribera et al., 2013; Juneja et al., 2022). In cirrhotic rats, high levels of pro-lymphangiogenic factors in the liver, such as VEGF-C and VEGF-D, have also been found to be positively associated with disease progression (Tugues et al., 2005). The density and diameter of gut LVs in human cirrhosis, however, remain elusive. Therefore, to characterize intestinal LVs in cirrhotic patients, we collected D2-biopsies from control and cirrhotic patients and immunostained with PDPN, a widely accepted marker of LVs presents on the surface of lymphatic endothelium (Figure 1A). PDPN stained sections were semi-quantitatively accessed and scored from 0–8. The diameter of LVs was also measured for dilation. Histologically, there was a weak and minimal PDPN positivity in the control biopsies. Patients with compensated and decompensated cirrhosis displayed a large number of PDPN+ dilated lymphatic channels, consisting of a single layer of endothelial cells in the mucosal and submucosal regions of D2-biopsies. Detailed calculation of PDPN scores in different patients is given in Supplementary Table S4. The mean PDPN scores in decompensated cirrhosis (6.91 ± 1.26) were significantly higher than in patients with compensated cirrhosis (3.25 ± 1.60, *p* < 0.0001, Figure 1B). We observed significantly dilated LVs in decompensated (193.1 ± 28.8 µm) and compensated (158 ± 11.1 µm) patients as compared to controls (138.5 ± 9.8 µm) (*p* < 0.05 each, Figure 1C). We have also checked the expression of key pro-lymphangiogenic factor VEGF-C protein in the D2-biopsies of control and cirrhotic patients and found significantly increased levels in decompensated (1.7 arbitrary unit (a.u.)) and compensated (0.9 a.u.) cirrhotic patients, compared to controls (0.6 a.u.) (*p* < 0.0043, Supplementary Figure S2). Along with this, we have also quantified the mRNA levels of some pro-lymphangiogenic factors, *FLT4, LYVE1*, and *PDPN*, in the D2-biopsies of control and cirrhotic patients. *LYVE1* (4-fold) and *PDPN* (9-fold) mRNA levels were elevated in patients with cirrhosis compared to controls (*p* < 0.0001, Supplementary Figure S2). We also compared PDPN scores in cirrhotic patients with and without different complications of decompensated cirrhosis. PDPN scores were significantly increased in patients with ascites and variceal bleeding *versus* those without ascites and bleeding (*p* < 0.05, Figure 1D, E). We did not find any significant difference in the PDPN scores of patients with and without HE (*p* = 0.13, Figure 1F).

### 3.3 PDPN score correlated with intestinal permeability and systemic inflammation

**FIGURE 2:**
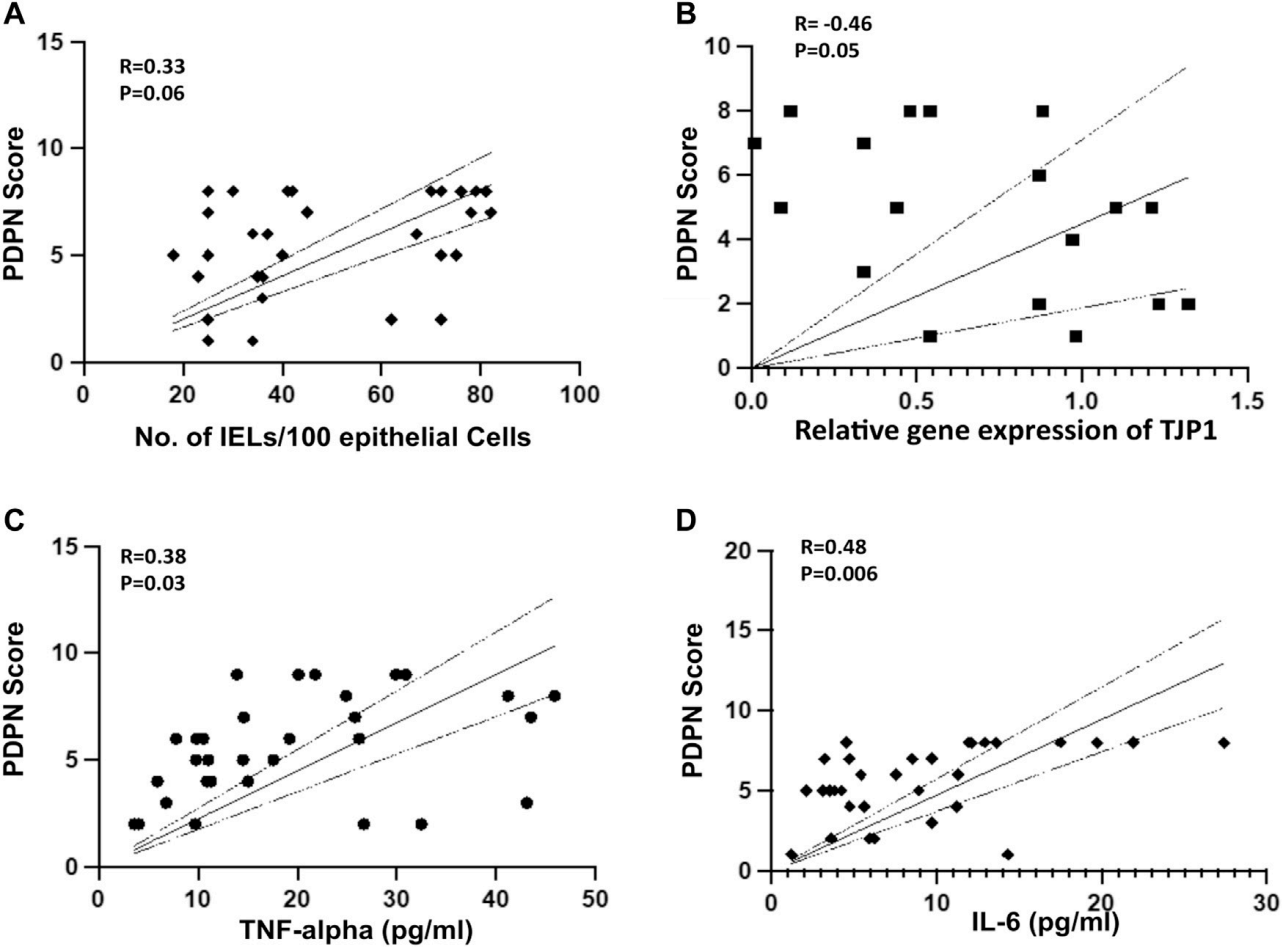
**(A)** Pearson’s Correlation between PDPN score and number of IELs in liver cirrhosis patients (R = 0.33, *p* = 0.06, *n* = 31) **(B)** Pearson’s Correlation between PDPN score and relative mRNA expression of *TJP1* (R = -0.46, *p* = 0.05, *n* = 20) **(C, D)** Pearson’s Correlation between PDPN score and plasma level of TNF-α (R = 0.38, *p* = 0.03) and IL-6 (R = 0.48, *p* = 0.006) in patients with liver cirrhosis (*n* = 31 each). Differences between groups were calculated by students’ unpaired *t*-test. Data represents mean ± standard deviation. The dotted lines represent 95% Confidence Interval (CI). R is correlation co-efficient. IEL: Intraepithelial lymphocytes.

We next investigated if PDPN scores are associated with intestinal inflammation and permeability in cirrhotic patients. Intestinal inflammation was characterized by the numbers of CD3^+^ duodenal intraepithelial lymphocytes (IELs), CD68^+^ macrophages, and villi structures in different study groups. The IELs were not significantly different between compensated and decompensated cirrhotic and also were not significantly correlated with PDPN scores ((Supplementary Figure S3, Figure 2A), r = 0.33, *p* = 0.06). CD68^+^ macrophages per field were found to be non-significantly increased in compensated (4.1 ± 1.16) patients compared to controls (2.4 ± 0.9, *p* = 0.09) but significantly elevated in decompensated cirrhotic patients (5.8 ± 2.0) compared to compensated or controls (Supplementary Figure S3, *p* < 0.05 each). Also, the villi: crypt ratio was 1:1 in the duodenum of decompensated cirrhotic with patchy mild blunting of villi as compared to 2:1 in control and compensated cirrhotic patients with no blunting of villi. In decompensated cirrhotic patients, there was also a depletion of goblet cells on the surface epithelium (Supplementary Figure S3). For intestinal permeability and inflammation, we examined the mRNA level of tight junction protein, ZO-1 (*TJP1*), Occludin (*OCLN*), *TNF-α,* and *IL-6* in some of the biopsy samples (Supplementary Figure S4). The expression of *TJP1* gene was significantly reduced in patients with decompensated cirrhosis compared to compensated cirrhosis (*p* < 0.05). A correlation analysis between *TJP1* gene expression and PDPN score revealed a significant negative correlation in the patients (r = -0.46, *p* = 0.05, Figure 2B). *OCLN* mRNA levels were significantly reduced in decompensated cirrhotic patients compared to controls (*p* < 0.05). Both *TNF*-*α* and *IL-6* mRNA levels were significantly increased in cirrhotic patients compared to control (*p* < 0.05 each). Next, we measured serum TNF-α and IL-6 levels to investigate the association of PDPN score with systemic inflammation. Serum TNF-α levels did not significantly differ between compensated and decompensated cirrhotic patients (Supplementary Figure S5). IL-6 levels, on the other hand, were significantly higher in patients with decompensated cirrhosis (Supplementary Figure S5, *p* = 0.02). High systemic levels of TNF-α (r = 0.38, *p* = 0.03) and IL-6 (r = 0.48, *p* = 0.006) significantly correlated with high PDPN scores in cirrhotic patients (Figure 2C, D).

### 3.4 PDPN score correlates with MELD and CTP scores of patients with cirrhosis

**FIGURE 3:**
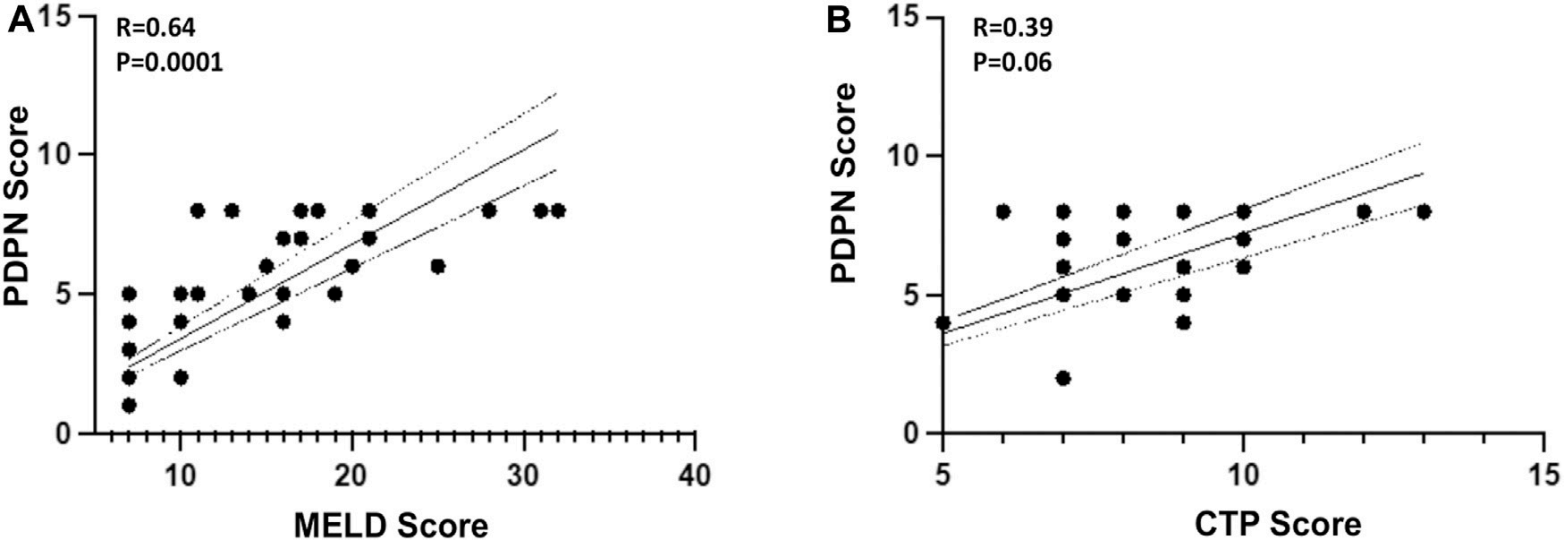
Pearson’s Correlation between PDPN score and **(A)** MELD Score (R = 0.64, *p* = 0.0001) **(B)** CTP Score (R = 0.39, *p* = 0.06) in liver cirrhosis patients (*n* = 24–30). Dotted lines represent 95% CI. R is correlation co-efficient. MELD: Model for end-stage liver disease; CTP: Child-Turcotte-Pugh.

Given a significant increase in the PDPN+ lymphatic channels in decompensated patients, we evaluated if the PDPN score correlates with liver disease severity. We performed a binary logistic regression analysis along with other clinical factors of decompensation. Cox regression was not performed as we did not have the data for the time of decompensation. The analysis showed that high levels of bilirubin and PDPN scores were relevant risk factors, and albumin was a protective factor (Supplementary Table S5). Multivariate logistic regression analysis excluded all these factors, and none of them emerged as a significant prognostic factor, suggesting a high correlation between these factors. The PDPN scores were significantly correlated with the Albumin (r = -0.62, *p* < 0.0001) bilirubin (r = 0.69, *p* < 0,001) and creatinine (r = 0.48, *p* = 0.005) in the cirrhotic patients. PDPN score also showed a correlation with both MELD (r = 0.64, *p* < 0.0001) and CTP scores (r = 0.39, *p* = 0.06) (Figure 3A, B). Although a definite relationship between the existing scores and PDPN was observed, variability was observed in certain patients. For example, patients with MELD scores of 18–20 had PDPN scores ranging from 4 to 8, and similarly for patients with CTP scores of 7 had PDPN scores from 2 to 8.

### 3.5 PDPN score associates with 3-month mortality in decompensated cirrhotic patients

**FIGURE 4:**
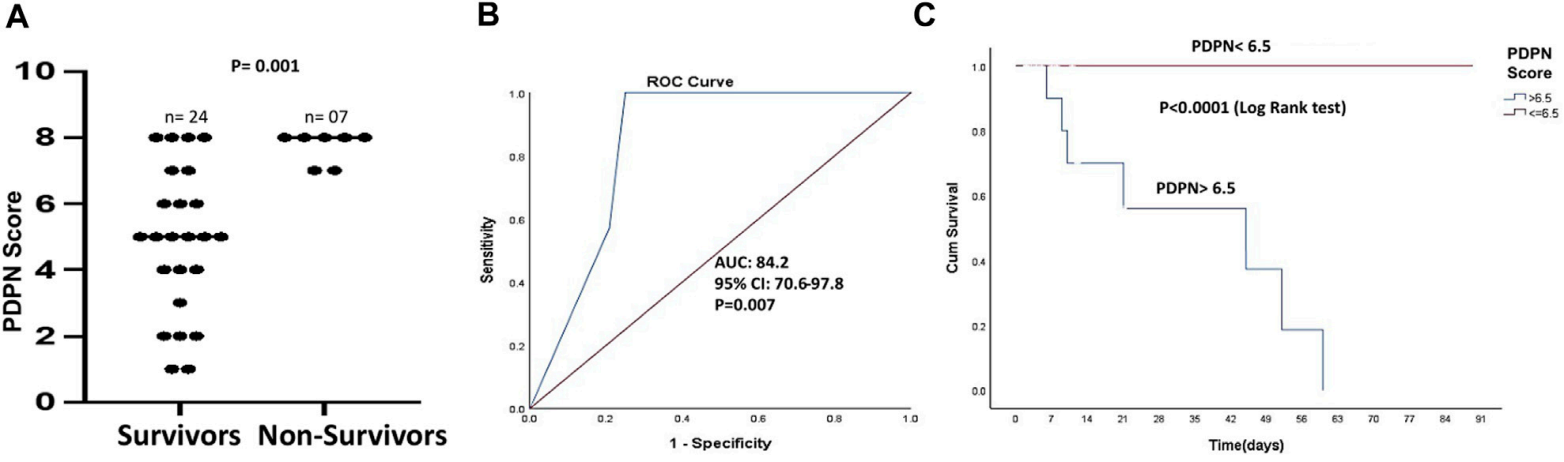
PDPN score as a mortality predictor in liver cirrhosis patients **(A)** Dot plots showing PDPN scores in survivors (*n* = 24) and non-survivors (*n* = 7) in patients with cirrhosis (*p* = 0.001) **(B)** ROC curve of PDPN score discriminating the survivors from the non-survivors (AUC = 84.2, *p* = 0.007) **(C)** Kaplan–Meier curve of survival with low and high PDPN score (cut-off value: 6.5), *p* < 0.0001 (log-rank analysis). Differences between groups were calculated by Mann-Whitney ‘U’ test. ROC: Receiver operating characteristics; AUC: Area under the curve.

We next studied if PDPN scores varied between the survivors and non-survivors. There was no mortality in patients with compensated cirrhosis within 3 months. Among patients with decompensated cirrhosis, 36.8% mortality was observed. PDPN scores were significantly different between the survivors and the non-survivors (Median values: 5 (1–8) vs. 7 (7–8), respectively, *p* = 0.001, Figure 4A). Next, we studied whether PDPN scores also predicted 3-month mortality in cirrhotic patients by Cox regression. Along with the laboratory parameters, we included the presence of complications such as bleeding, varices, ascites, and HE in this analysis. The univariate analysis showed several laboratory parameters and complications as significant predictors of mortality, including high bilirubin, creatinine, INR, presence of variceal bleeding, jaundice, HE, and oesophageal varices. High CTP, MELD, and PDPN scores also emerged as significant predictive factors of mortality in the univariate analysis (Table 2). In multivariate analysis, we excluded the clinical parameters that were a part of the severity scores and included the presence of variceal bleeding, CTP, MELD, and PDPN score for analysis. PDPN score emerged as a significant and independent mortality predictor (Table 2). The ROC curve for PDPN score showed an AUC of 84.2 (95% CI: 70.6–97.8, *p* < 0.007, Figure 4B). The cutoff value of PDPN scores for predicting significant 3-month mortality was above 6.5, with a sensitivity of 100% and a specificity of 75%. With the obtained cutoff values of PDPN, we also constructed survival curves in cirrhotic patients with high and low PDPN scores in liver cirrhosis using the Cox regression. The log-rank analysis showed that mortality in patients with a PDPN score less than equal to 6.5 was significantly lower at 3 months than in patients with a PDPN score greater than 6.5 (Figure 4C, Chi-Square: 20.5, *p* < 0.0001). There was no death in cirrhotic patients with a PDPN score less than 6.5, while 54% of patients (all decompensated) died within 3 months, which had a PDPN score greater than 6.5. The cause of death in all patients was septic shock and multiple organ failure.

**TABLE 2:** Predictive factors for 3-month mortality in patients with cirrhosis (N = 31).

	Univariate analysis	
Risk factor	Or (95% CI)	*p*-value
Age	0.99 (0.91–1.06)	0.79
Sex	2.02 (0.24–16.88)	0.51
Globulin	0.58 (0.22–1.49)	0.26
TLC	1.04 (0.98–1.10)	0.17
Albumin	0.51 (0.21–1.25)	0.15
Bilirubin	1.12 (1.03–1.22)	0.006*
Sodium	0.88 (0.76–1.01)	0.08
AST^#^	3.1 (0.86–11.2)	0.08
ALT^#^	1.54 (0.39–6.10)	0.53
INR	3.24 (1.19–8.80)	0.05*
Creatinine	18.67 (2.42–144.10)	0.005*
Platelet	0.99 (0.98–1.01)	0.81
Variceal Bleeding	5.09 (1.12–23.02)	0.03*
Esophageal Varices	4.39 (0.96–19.99)	0.06*
Ascites	386.1 (0.22–654354)	0.116
HE	9.43 (1.61–55.1)	0.01*
CTP	1.59 (1.13–2.23)	0.007*
MELD	1.16 (1.04–1.29)	0.006*
PDPN Score	6.01 (1.17–30.74)	0.03*
Multivariate Analysis (with scores and presence of variceal bleeding)		
PDPN Score	5.61 (1.08–29.109)	0.04*

**‘** *#'* Log values of these parameters were taken. ‘*’ denotes significant p values (Cox regression). Significance was taken as p< 0.05. OR: odd ratio; CI: Confidence Interval. TLC: total lymphocyte count; HE: hepatic encephalopathy; MELD: Model for end-stage liver disease; CTP: Child-Turcotte-Pugh.

## 4 Discussion

Inflammation-induced remodeling of the lymphatic network occurs by vascular endothelial growth factor (VEGF)-A/C/D signaling through VEGF receptor 2/3 (VEGFR-3). Besides these VEGFs, PDPN has also been reported to have significant effects on the proliferation, migration, and tube formation of lymphatic endothelial cells (Navarro et al., 2008; Navarro et al., 2011). Our study reports that patients with cirrhosis have a significantly increased number of lymphatic channels characterized by PDPN and VEGF-C immunostaining and *PDPN* and *LYVE1* expression in the D2-biopsy lysates. Immunostaining with PDPN demonstrated enlarged and dilated lymphatic channels in decompensated cirrhosis patients compared with compensated ones. Patients with and without HE did not differ significantly for PDPN score, possibly because all decompensated patients had ascites, to begin with, and HE was a second decompensation event present only in some patients.

An increased number of dilated PDPN+ intestinal LVs in cirrhotic patients signifies impaired lymph drainage and compensatory lymphangiogenesis. Compared with the lymphatic flow in healthy subjects (1L/day), studies have demonstrated a significant increase in abdominal lymph production (30-fold) and lymph flow in the thoracic duct of cirrhotic patients with ascites (eight to nine L/day) (Vollmar et al., 1997). The most common cause of ascites in cirrhosis is elevated pressure in the portal circulation, causing hypertension. A positive correlation exists between lymph production in the gut and lymph flow with increasing portal pressures (Megevand, 1969). Gut LVs are capable of draining a moderate amount of lymph and returning it to the systemic circulation, preventing the accumulation of fluid in the abdominal cavity. But as cirrhosis and portal hypertension progresses, the vascular dysfunction or hyperdynamic systemic and splanchnic circulation increases the mesenteric blood flow and portal pressure, which in turn, causes more fluid to escape from the vessels. This excess fluid greatly enhances the production and flow of interstitial hepato-intestinal lymph leading to fluid loss in a significant amount from the peritoneal lining and serosal surfaces of the bowel (Oikawa et al., 1998; Aller et al., 2010). Concerning intrahepatic LVs, it is known that there is an increase in the number of dilated D2-40/podoplanin liver LVs and lymphangiogenesis in patients with cirrhosis and portal hypertension, regardless of disease etiology (Oikawa et al., 1998; Yokomori et al., 2010; Ma et al., 2021). Hence, it might be deduced that there is an increased production of intestinal and hepatic lymph due to ongoing inflammation in the gut-liver axis and vascular blood flow changes. The overwhelmed and defective lymphatic flow and drainage mechanism leads to the accumulation of ascites.

PDPN scores significantly correlated with many clinical parameters of cirrhosis, including the severity scores, MELD, and CTP in our patients, suggesting the utility of PDPN scores in assessing disease severity. However, it is to be noted that many patients with a particular value of MELD or CTP score showed a range of values for the PDPN score, indicating that the variables are different. We also observed an inverse correlation between the expression of *TJP1*, an intestinal permeability gene, and PDPN scores in our patients, underscoring the contribution of increased intestinal permeability in LVs remodeling. Gut dysbiosis with increased intestinal permeability is a characteristic feature of cirrhosis (Ponziani et al., 2018). Intestinal permeability has been reported to be significantly higher in decompensated cirrhosis patients with ascites and encephalopathy compared with compensated cirrhosis (Pascual et al., 2003). Patients with decompensated cirrhosis also have significantly reduced expression of tight junction proteins in the D2-biopsies as compared to compensated (Assimakopoulos et al., 2012). With increased intestinal epithelial permeability, gut LVs are exposed to increased fluid and pathogen load in cirrhosis that might be causing LVs remodeling, dilation, and lymphangiogenesis, as observed in intestinal inflammation (Stephens et al., 2019).

In our study, a higher PDPN score was identified as an independent predictor of 3-month mortality when MELD, CTP, and the presence of variceal bleeding were included in the multivariate analysis. This signifies the importance of dilated intestinal lymphatic channels in predicting mortality in cirrhotic patients along with the MELD and CTP scores. This also calls for further prospective studies to determine the use of PDPN scores for organ allocation to reduce mortality in patients on the liver transplantation waiting list. Increased mortality due to lymphatic abnormality may be attributed to its role in inducing systemic inflammation. Intestinal hyperpermeability is associated with an increase in pathological bacterial translocation in cirrhosis. Gut bacteria and other endotoxins use the mesenteric collecting lymph vessels and drain into the mesenteric lymph nodes that serve as key points to handle the bacterial load in the gut and prevent their transfer in the systemic circulation (Macpherson and Smith, 2006). Impaired lymph drainage and persistent lymph stasis in the intestine could contribute to chronic inflammation by reducing the drainage of bacteria and bacterial products to the lymph nodes. Gut inflammation in cirrhosis might also produce profound changes in the mesenteric lymph composition. Elevated endotoxins and inflammatory cytokines are found in the mesenteric lymph and thoracic duct before they appear in the portal vein (Deitch, 2002). In fact, mesenteric lymph can be termed as a splanchnic vehicle for systemic proinflammatory responses. Disruption of intestinal lymphatics and lymph nodes has earlier been shown to cause systemic infection, massive inflammatory responses, and mortality (Jang et al., 2013). A pathogenic role of mesenteric lymph has also been suggested in multiple organ dysfunction. It has been reported that although there were no live bacteria in the thoracic duct lymph, the levels of lymph inflammatory cytokines were higher in ICU patients with multi-organ dysfunction as compared to those without organ dysfunction (Lemaire et al., 1999; Deitch, 2002). It has been postulated that during gut inflammation and infection, mesenteric lymph with toxic factors potentiate distant organ failure, especially lung failure, by exaggerating the systemic inflammatory responses, even when these translocating bacteria do not reach the systemic circulation (Ma et al., 2021). High PDPN scores in our patients were significantly associated with both systemic TNF-α and IL-6 levels and also IELs in the D2-biopsies, suggestive of both gut and systemic inflammatory immune responses in patients with dilated lymphatic channels. Systemic inflammation, as reflected by plasma IL-6 levels, is a valuable biomarker of advanced chronic liver disease progression. IL-6 predicts the risk of the first decompensation in patients with compensated cirrhosis and also 1-year liver-related mortality predictor or the need for liver transplantation in those with decompensated cirrhosis (Costa et al., 2021). It would be worthwhile to study the levels of IL-6 and other inflammatory cytokines in mesenteric lymph of patients with cirrhosis with varying severity and correlate them with duodenal PDPN scores and mortality.

To summarize, we report here that an increased number of dilated LVs, characterized by PDPN expression in the D2-biopsies, are a characteristic feature of patients with decompensated cirrhosis and ascites and PDPN score serves as a valuable predictor of 3-month mortality. Future studies with a larger sample size to confirm the prognostic value of PDPN score across stages of cirrhosis are warranted. Further understanding of whether gut lymphangiogenesis is a pathological or protective mechanism in cirrhosis would help us to design specific therapeutic interventions for such patients.

## Data Availability

The original contributions presented in the study are included in the article/Supplementary Material, further inquiries can be directed to the corresponding author.
