# Supplementary material for: Podoplanin-positive dilated lymphatic vessels in duodenum associates with three-month mortality in patients with cirrhosis
Source: Front Physiol. 2023 May 25;14:1045983. doi: 10.3389/fphys.2023.1045983 (PMC10248415; doi:10.3389/fphys.2023.1045983)
Supplement: Supplementary file 1 [file DataSheet1.pdf]

Supplementary Material

Table of Content:

Definition and Methods …………………………………………………………………………...2

Supplementary tables …………………………………………...3

Supplementary figures and legends………………………………………………………………..8

References………………………………………………………………………………………....14

Definition and calculation of important parameters and regression methods:

MELD Score: MELD stands for "Model for end-stage liver disease." This score is used for predicting mortality in patients with end stage liver disease (Singal and Kamath, 2013).

Calculation: 9.57 × log (creatinine) + 3.78 × log (total bilirubin) + 11.2 × log (INR) + 6.43.

CTP Score: Child–Pugh score is used to assess the prognosis of chronic liver disease (Assimakopoulos et al., 2012).

Calculation: CTP score is obtained by adding the score for each parameter:

	**Points***		
	**1**	**2**	**3**
Encephalopathy	None	Grade 1-2<br>(or precipitant-induced)	Grade 3-4<br>(or chronic)
Ascites	None	Mild/Moderate<br>(diuretic-responsive)	Severe<br>(diuretic-refractory)
Bilirubin (mg/dL)	< 2	2-3	> 3
Albumin (g/dL)	> 3.5	2.8-3.5	< 2.8
PT (sec prolonged)<br>or INR	< 4<br>< 1.7	4-6<br>1.7-2.3	> 6<br>> 2.3

CTP class:

A = 5-6 points

B = 7-9 points

C = 10-15 points

Cox Regression Model: Cox regression is a method to analyze the effect of variables under consideration upon the time a specified event will happen. The method assumes that the effects of the predictor variables upon the happening of the event are constant through time and are additive in one scale.

Supplementary Tables

Supplementary Table 1

Primer sequence used in performing RT-PCR.

Gene	Forward Sequence	Reverse sequence
FLT4	GGCAGCTTCTCGCAGGTGT	GTTGGGGTCATGGGGAATTCCT
LYVE1	GCCTGTAGGTGCTGGGACTAAG	CCCAGCAGCTTCATTCTTGAATG
PDPN	GTGGATGGAGACACACAGACA	GCGAGTACCTTCCCGACATT
TJP1	GAATGATGGTTGGTATGGTGCG	TCAGAAGTGTGTCTACTGTCCG
OCLN	ATGAGACAGACTACACAACTGG	TTGTATTCATCAGCAGCAGC
TNF-alpha	GCCCAGGCAGTCAGATCATCT	TTGAGGGTTTGCTACAACATGG
IL-6	GCAACACCAGGAGCAGCC	AACTCCTTCTCCACAAGCGC

Supplementary Table 2: Details of the primary antibody used

**Antibody name**	**Reactivity**	**Host**	**Company**	**Catalogue Number**
Podoplanin	Human, Mouse, rat	Rabbit	Invitrogen, United States,	PA5-37285
CD3	Human		PathnSitu Biotechnologies	PP160
CD68	Human		PathnSitu Biotechnologies	PM113
VEGFC	Human	Mouse	Invitrogen, Unites States	MA5-26494

Supplementary Table 3:

Podoplanin (PDPN) Scoring System of lymphatic vessels:

**Intensity of stained area (A)**	**Percentage proportion of PDPN+ stained area/field (B)**	**Final PDPN Score (A+B)**
0= none	0= 0-5%	0 (Lowest Score)
1= 1-25%	1= 6–25%	2
2= 25-50%	2= 26–50%	4
3= 50-75%	3= 51–75%	6
4= 75-100%	4= 76-100%	8(Highest Score)

Supplementary Table 4

Calculation of total pdpn score of patients with cirrhosis

**Patient Groups**	**Complication**	**Intensity of pdpn+ vessels**	**Density of pdpn+ vessels**	**Total Pdpn Score**
Compensated Cirrhosis (n=12)	-	1.75 ± 0.75	1.5 ± 1	3.25 ± 1.6
Decompensated Cirrhosis (n=19)	Ascites	3.36 ± 0.8	3.57 ± 0.69	6.9 ± 1.26
	HE	3.3 ± 1.25	3 ± 1.24	6.3 ± 2.35
	Non-HE	2.61 ± 1.07	2.42 ± 1.3	5 ±2.30
	Bleed	3.75 ± 0.46	3.25 ± 0.7	7 ± 1.06
	Non-Bleeder	2.95 ± 1.1	2.2 ± 1.25	5.15 ± 2.22

Data is given as mean ± SD

Supplementary Table 5

Clinical Variables Associated with Cirrhosis

	**Univariate Analysis**	
**Risk Factor**	**OR (95% CI)**	**P value**
Age	0.99 (0.92-1.07)	0.94
Sex	0.30 (0.02-3.96)	0.37
Globulin	1.06 (0.41-2.74)	0.89
TLC	1.02 (0.90-1.16)	0.65
Albumin	0.24 (0.06-0.86)	0.02*
Bilirubin	3.34 (1.08-10.29)	0.03*
Sodium	0.86 (0.70-1.07)	0.18
AST[#]	5.94 (0.99-35.5)	0.05
ALT[#]	1.58 (0.98-2.06)	0.21
INR	21.11 (0.73-610.5)	0.07
Creatinine	32.05 (0.94-1090.1)	0.06
Platelet	0.98 (0.97-0.99)	0.06
Pdpn Score	7.28 (1.28-41.46)	0.02*

'#' Log values of these parameters were taken. '' denotes significant p values (Binary logistic regression). Significance was taken as P<0.05. OR: Odds Ratio; CI: Confidence Interval. TLC: Total lymphocyte count; MELD: Model for end-stage liver disease; CTP: Child-Turcotte-Pugh.*

Supplementary Figures

(A)

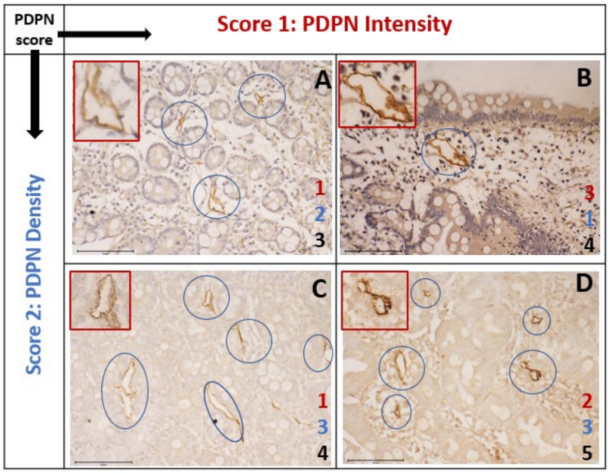

Supplementary Figure 1: PDPN scoring based on sum of two parameters namely (1) intensity (enlarged image in red) and (2) density (encircled with blue) of PDPN positive stained LVs on the scale from 0-4 each. Numbers in red and blue represents intensity and density score respectively. Number in black represent total PDPN score derived from sum of (1) Intensity + (2) Density of PDPN positive LVs. LVs: Lymphatic Vessels; PDPN: podoplanin. Scale Bar: 100µM each.

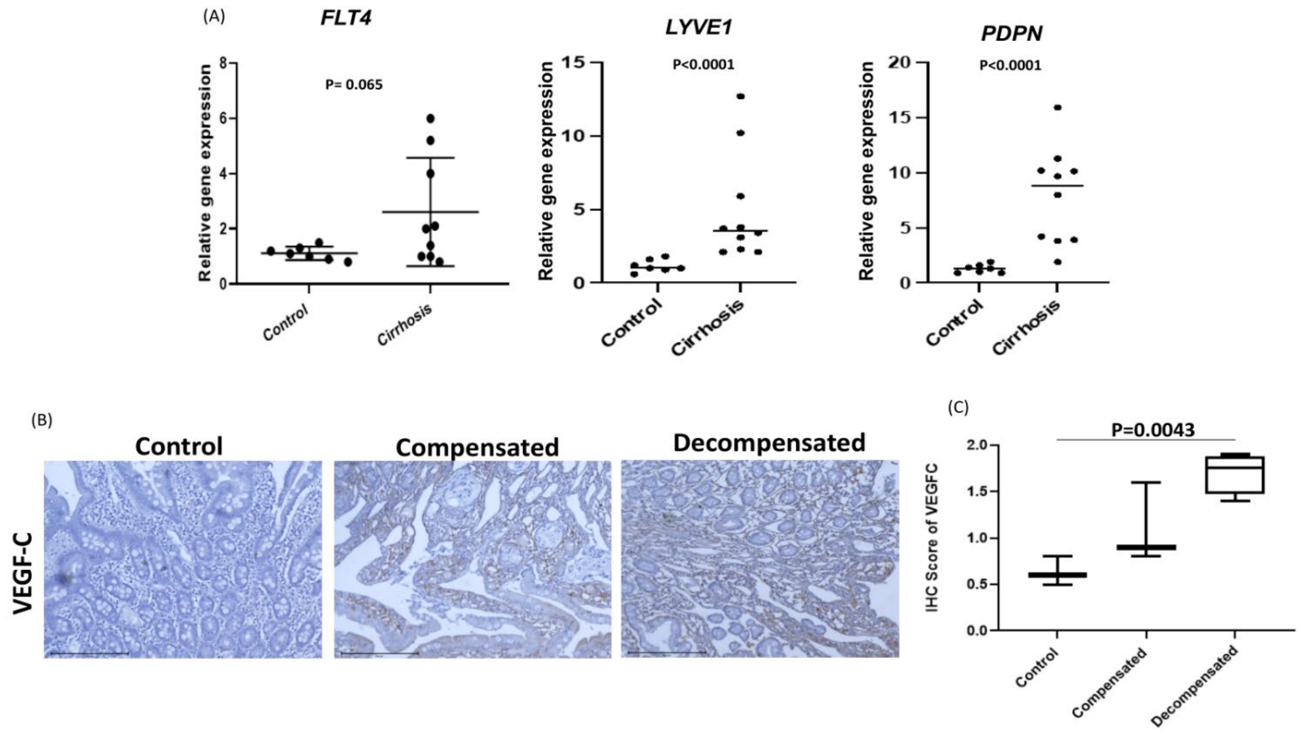

Supplementary Figure 2: (A) Representative image of D2-biopsies showing expression of pro-lymphangiogenic factor, VEGFC in control, compensated and decompensated cirrhotic patient. Scale Bar: 200µM. **(B)** Bar graph showing quantification of VEGFC expression in control (n=9), compensated (n=12) and decompensated (n=19) cirrhotic patients. Differences between groups were calculated by Mann-Whitney 'U' test. **(C)** Expression of LVs markers, *FLT4*, *LYVE1* and *PDPN* in D2-biopsies of controls (n=7) and liver cirrhosis patients (n=10). Dot plots showing relative gene expression determined by quantitative real-time PCR in controls and patients with liver cirrhosis. Differences between groups were calculated by Mann-Whitney 'U' test. D2: Duodenal; VEGFC: Vascular Endothelial Growth Factor C.

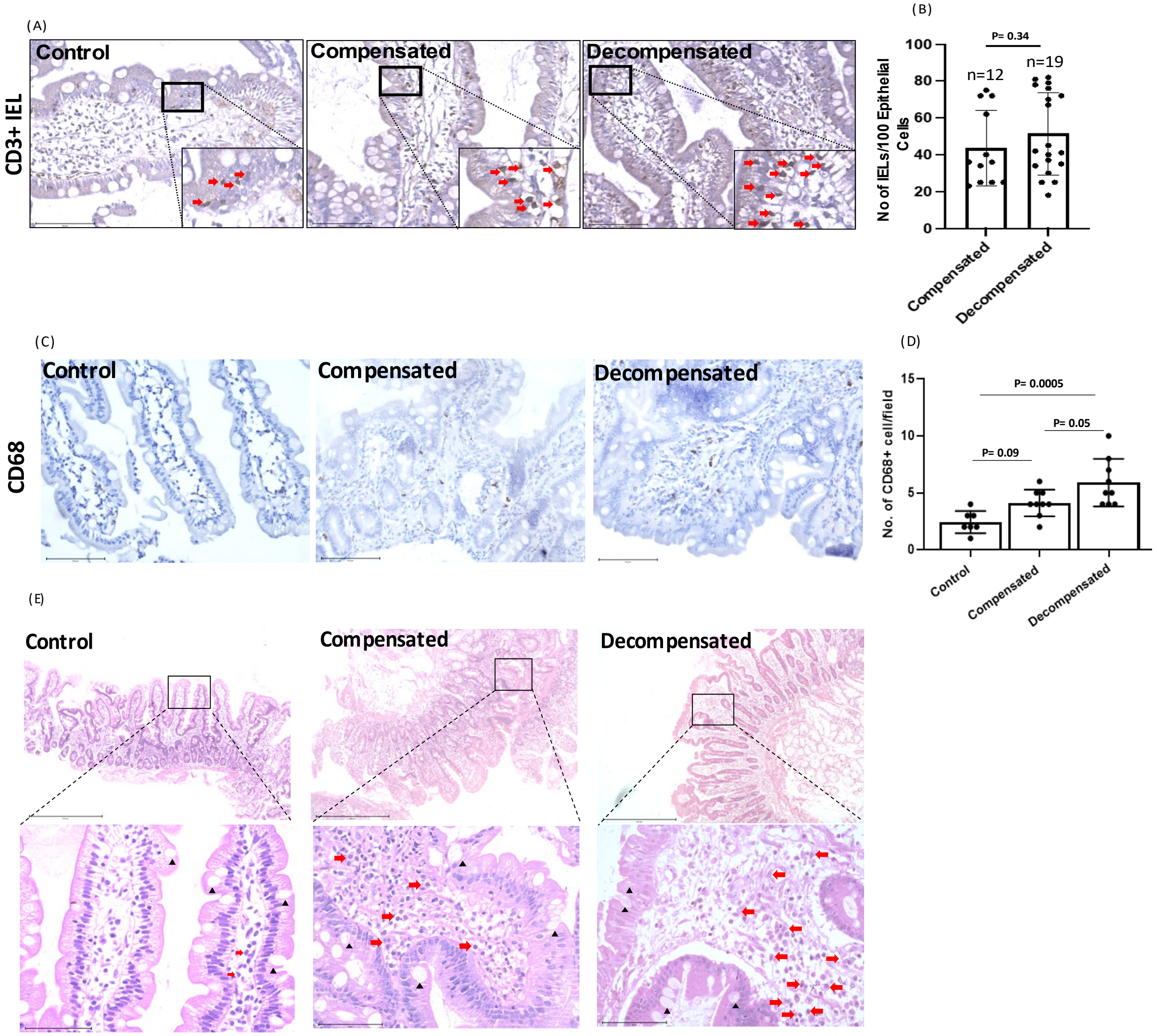
(A)
CD3+ IEL
Control
Compensated
Decompensated
(B)
P= 0.34
n=12
n=19
No of IELs/100 Epithelial Cells
100
80
60
40
20
0
Compensated
Decompensated
(C)
CD68
Control
Compensated
Decompensated
(D)
P= 0.0005
P= 0.05
P= 0.09
No. of CD68+ cell/field
15
10
5
0
Control
Compensated
Decompensated
(E)
Control
Compensated
Decompensated

Supplementary Figure 3: (A) Representative image for CD3+ stained IELs in D2-biopsy sections of control, patients with compensated and decompensated cirrhosis. Enlarged images of selected areas are given as inset. Scale Bar: 100µM. **(B)** Bar graph showing number IELs/100 epithelial cells in patients with compensated (n=12) and decompensated cirrhosis (n=19). Differences between groups were calculated by student's unpaired 't' test. **(C)** Representative image for CD68+ stained macrophages in D2-biopsy sections of control, patients with compensated and decompensated cirrhosis. Scale Bar: 100µM. **(D)** Quantification of CD68+ cells per field in D2-biopsies of control (n=7), compensated (n=9) and decompensated (n=9) cirrhotic patients. Differences between groups were calculated by Mann-Whitney 'U' test. **(E)** Villi anomalies in D2-biopsies of different study groups such as length and blunting. Black arrow indicates goblet cells and red arrow indicate neutrophil infiltration in control, compensated and decompensated patients. IEL: Intraepithelial lymphocytes; D2: duodenal. Scale Bar: 500µM upper panel, 75µM lower panel**.**

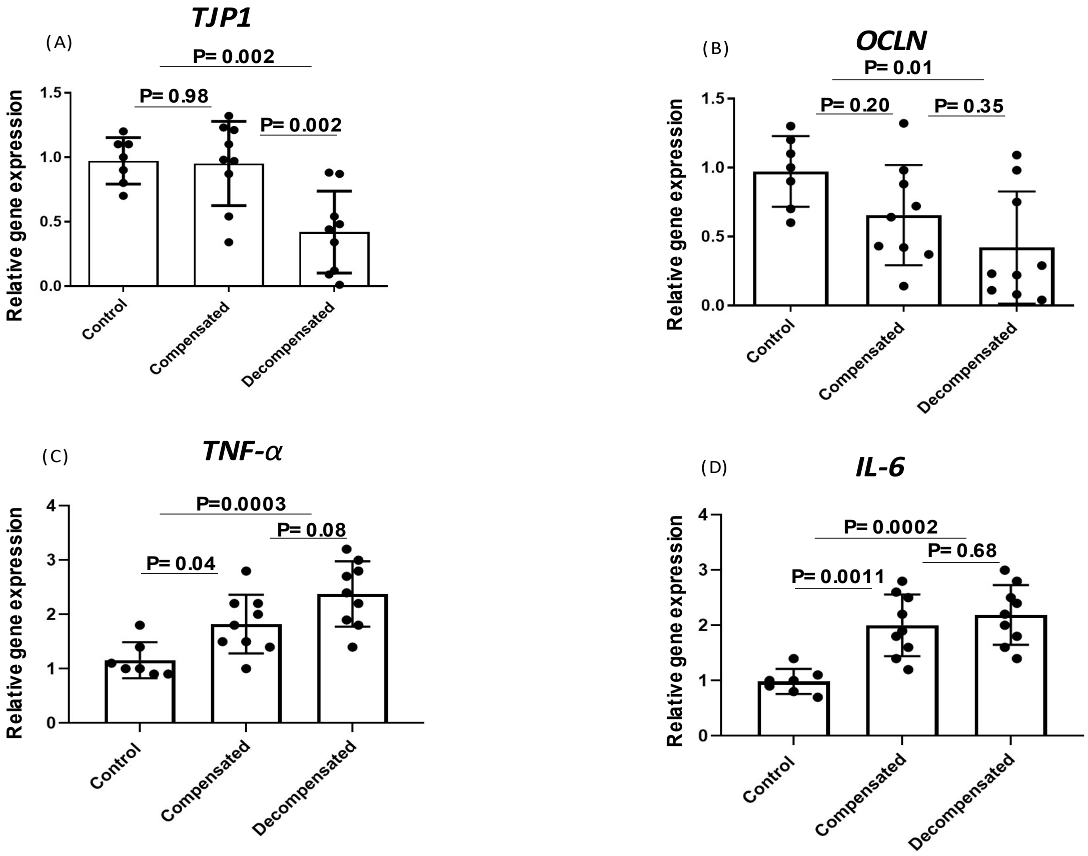

Supplementary Figure 4: Bar graph showing relative mRNA expression of **(A)** *TJP1* **(B)** *OCLN* **(C)** *TNF-α* and **(D)** *IL-6* in D2-biopsies of control (n=7) compensated (n=9) and decompensated (n=9) cirrhotic patients. Differences between groups were calculated by Mann-Whitney 'U' test

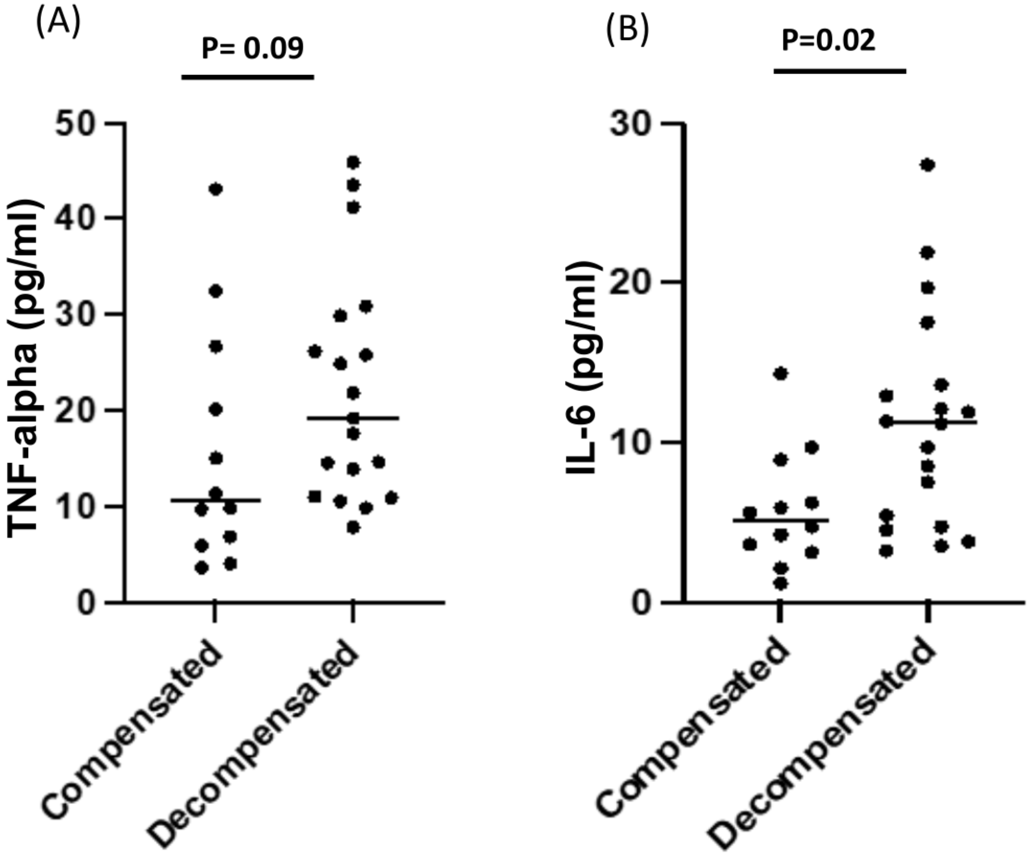

Supplementary Figure 5: Dot plots showing **(A)** TNF-α and **(B)** IL-6 levels in serum of patients with compensated (n=12) and decompensated cirrhosis (n=19). Differences between groups were calculated by Mann-Whitney 'U' test

References:

1. Assimakopoulos, S. F., Tsamandas, A. C., Tsiaoussis, G. I., Karatza, E., Triantos, C., Vagianos, C. E., et al. (2012). Altered intestinal tight junctions' expression in patients with liver cirrhosis: a pathogenetic mechanism of intestinal hyperpermeability. *Eur. J. Clin. Invest.* 42, 439–446. doi: 10.1111/J.1365-2362.2011.02609.X.
2. Singal, A. K., and Kamath, P. S. (2013). Model for End-stage Liver Disease. *J. Clin. Exp. Hepatol.* 3, 50. doi: 10.1016/J.JCEH.2012.11.002.
